# Case of Fibrous Tumor Removed from the Broad Ligament of Uterus

**Published:** 1867-12

**Authors:** Walter Burnham

**Affiliations:** Lowell, Mass.


### CASE OF FIBROUS TUMOR REMOVED FROM THE BROAD LIGAMENT OF UTERUS.

By Walter Burnham, M.D., of Lowell, Mass.

Reported by CHAS. M. CLARK, M.D., Chicago, Ill.

History of Case.—Mrs. J. L. H., aged 50 years. She had enjoyed uninterrupted good health for the preceding forty-two years of her life, and had borne four children; only one of the number is now living. In 1858, she suffered from a severe attack of "bilious-remittent fever;" the sequelæ of which was, as she states, general anasarca, which yielded readily to the treatment with cream of tartar; and during the years 1859 and 1860, was comparatively well, with the exception of occasional hot flushes over the surface of the body, at which time, the skin would become very red, and a profuse perspiration break out, succeeded by extreme prostration.

In September, 1861, she first noticed a small tumor in the right groin, (she had not been regular with her catamenia for the preceding five months, and her last child was then 13 years old,) in size like a walnut. It could be distinctly felt, and was moveable to great extent. She commenced bloating a great deal at this time, but still was able to perform the necessary duties of her home.

In November of the same year, she was attacked with peritoneal and ovarian inflammation, and was confined to her bed until the following February, 1862. After this time, the tumor and the bloating seemed to decrease, and the tumor changed from the right side to a position more noticed, over the umbilical region. From this time, and until the following September, she felt quite comfortable, and was able to perform her usual duties. At this time, she visited Chicago, and consulted with Prof. DeLaskie Miller, and the late Dr. Orrin Smith. They advised her to return to her home in Missouri, and leave the tumor alone.

After this, she had no more trouble until August, 1865, when she was prostrated with dysentery. She recovered, but did not regain her normal strength. Soon after, she removed to Chicago. In December, 1865, ascites was first noticed, and its accumulation was slow, and she consulted and remained under the treatment of an electrical doctor, who promised a cure by the "laying on of hands." Her statement is, that the "prestigitateur" of electropathy benefited her general health, but his treatment increased the size of the tumor, and the effusion within her abdomen.

At the time when dropsical effusion commenced, her skin became scaly, and looked like the scales of a fish when viewed transversely with the axis of her body. The tumor gradually enlarged from this time, and the effusion within the cavity was such as to necessitate the operation of paracentesis, which was performed June 12th, 1866, by Dr. David Dodge, of this city, under whose care she then was, and ten quarts of light straw-colored serum removed.

At the time Dr. Dodge assumed charge of the case, the abdomen was greatly distended; there was constant vomiting—nothing would remain in the stomach; rapid and weak pulse, and great emaciation. The urine was voided once in forty-eight hours, but scantily and very high colored. No appetite; would drink gruel, and vomit it immediately; skin hot and very dry. The treatment given at this time was blister applied to the abdomen and dressed with morphia, together with injections of morphia. She had, previously to the visit of Dr. Dodge, taken elaterium from a German physician on the west side.

I was called to see the case about the 14th day of July, 1866, in company with Dr. Dodge, and took away twelve (12) quarts of fluid. After the evacuation of the serum, I could grasp a large, nodulated tumor, about the size of a fœtal head, which was moveable, and would easily slide from one side to the other. Manipulation of the tumor gave her no pain. My prescription at this time was chlorate potass., tinct. ferri. mur., and ol. morrhuæ. August 14th, I again tapped her, and took away fourteen quarts of a straw-colored serum; also, September 8th, when fourteen quarts was voided; and again, October 1st, when I took away fourteen quarts. An operation for the removal of the tumor was now seriously thought of, and the woman's consent gained, and the day appointed; but on consultation with several eminent physicians, who visited the case with me, it was thought best to defer the operation until she should gain more strength.

October 3d, I made a close and thorough examination; found that the tumor had increased in size, was more nodulated, and that the effusion was increasing rapidly.

October 17th, I again tapped her; drew off sixteen quarts of fluid, the last pint of which was mixed with blood, but this was due to contact of the tumor with the canula, in her efforts in pressing with her hands to squeeze out the last drop from the abdominal cavity.

Soon after this, I left the city, and the case was assigned to

Dr. Seeley, who cared for it until the following operation by Dr. Burnham.

She bore the operation well, and slept soundly during the night without an opiate. No especial treatment seemed to be necessary after the removal of the tumor, except simple dressing to the wound, and nourishment.

During the night, she perspired very freely, the first action of the skin she had had since the commencement of the tumor.

The wound healed rapidly; and there has been no effusion within the abdomen up to the present time, October 18th, 1867. She is now enjoying good health and doing her own house work.

The tumor weighed three pounds and four ounces.

